# Phenotypic Diversity Analysis and Comprehensive Evaluation of Foxtail Millet Germplasm Resources Evaluated in Northern Xinjiang

**DOI:** 10.3390/plants15203158

**Published:** 2026-10-15

**Authors:** Yun Zhao, Jun Liu, Xiangwei Hu, Hui Wang, Hong-Jin Wang, Guojun Feng, Zaituniguli Kuerban

**Affiliations:** Crop Research Institute, Academy of Agricultural Sciences of Xinjiang Uyghur Autonomous Region, Urumqi 830091, China

**Keywords:** foxtail millet, germplasm resources, phenotypic traits, phenotypic diversity, principal component analysis, cluster analysis

## Abstract

Foxtail millet (*Setaria italica* L.) is an important functional food crop with high photosynthetic efficiency and drought tolerance, and it plays a significant role in arid and semi-arid agricultural systems. However, germplasm resources from Xinjiang remain inadequately characterized, limiting their use in breeding programs. This study systematically evaluated 203 foxtail millet germplasm accessions using 8 qualitative traits and 13 quantitative traits across two growing seasons (2023–2024) in northern Xinjiang. Phenotypic variation and diversity, trait correlations, and phenotypic grouping were analyzed using the Shannon–Weaver diversity index (H′ = −Σ p_i_ ln p_i_), coefficient of variation (CV), Pearson correlation, principal component analysis (PCA), and hierarchical cluster analysis. Among qualitative traits, leaf posture during flowering exhibited the highest phenotypic diversity index (H′ = 1.072), whereas leaf sheath color showed the lowest (H′ = 0.359). Predominant phenotypes included green seedling leaf color (77.83%), semi-erect seedling leaf posture (57.14%), drooping leaf posture during flowering (50.74%), curved panicle neck (74.88%), spindle-shaped panicle (83.25%), and yellow grain color (84.73%). Among quantitative traits, grain weight per panicle (GWE; CV = 38.91%), single panicle weight (SEW; CV = 37.65%), and yield (CV = 37.31%) showed the highest variability. Yield was strongly and positively correlated with SEW (r = 0.925, *p* < 0.001) and GWE (r = 0.925, *p* < 0.001), and SEW and GWE were almost collinear (r = 0.996). PCA of 12 standardized traits (SEW excluded to avoid redundancy with GWE) extracted five components explaining 65.55% of total phenotypic variation. Hierarchical clustering of the unstandardized trait means (yield in kg·mu^−1^), in which yield contributed most strongly to group separation, divided the accessions into four groups: Group I (129 accessions, 63.55%) was intermediate (mean yield 3532 kg·ha^−1^); Group II (37 accessions, 18.23%) had the lowest yield (1650 kg·ha^−1^) and panicle weights; Group III (32 accessions, 15.76%) had high yield (5282 kg·ha^−1^); and Group IV (5 accessions, 2.46%), all breeding lines, had the highest yield (6814 kg·ha^−1^), the heaviest panicles, and the thickest stems. The evaluated foxtail millet germplasm showed substantial phenotypic diversity under irrigated conditions at a single site, providing candidate materials for yield improvement and for further evaluation of stress resistance and adaptation. Group III and Group IV accessions are candidate parents for high-yield breeding; however, this requires validation in multi-location trials because only one irrigated site was evaluated and lodging and drought responses were not measured. Entry-mean repeatability of accession means across the two seasons ranged from 72.1% to 99.99%, and these values are likely upper bounds because genotype × year interaction could not be separated from residual error. These findings provide a phenotypic baseline for germplasm utilization in Xinjiang foxtail millet improvement programs.

## 1. Introduction

Foxtail millet (*Setaria italica* L.), belonging to the tribe Paniceae of the Poaceae family, is an annual self-pollinated crop that serves as an important functional food crop in the dry-farming areas of northern China [1]. After dehulling, it becomes millet and has been cultivated for approximately 10,000 years, making it one of the oldest domesticated crops [2]. Foxtail millet possesses several advantageous characteristics, including high C4 photosynthetic efficiency, exceptional drought tolerance, efficient nutrient utilization, and adaptability to poor soils, making it a strategic crop for sustainable agriculture in arid and semi-arid regions [3]. The grains are rich in fat, protein, B-group vitamins, and mineral elements such as calcium and magnesium, offering both nutritional and health-promoting benefits [4]. Its outstanding nutritional profile has earned it the designation as the “king of the five grains” in traditional Chinese agriculture [5]. Under the dual pressures of global cultivated land reduction and continuously increasing food demand, breeding breakthrough varieties with high yield, superior quality, stress resistance, and wide adaptability has become a core challenge for ensuring food security [6,7]. Foxtail millet, with its inherent climate-resilient characteristics, has gained renewed attention as a model for studying stress tolerance mechanisms in C4 cereals [8,9]. The availability of the reference genome sequence has facilitated molecular studies, yet the full breeding potential of foxtail millet germplasm remains largely untapped [10]. This potential includes, for example, improvement of drought tolerance, nutritional quality, and yield stability, each of which requires systematic phenotypic and genomic characterization.

Germplasm resources represent the essential foundation for crop improvement and genetic investigations [11]. These resources preserve phenotypic and genetic diversity and contain beneficial alleles associated with critical traits, including drought tolerance, disease resistance, early maturity, and improved grain quality [12]. Systematic assessment and characterization of germplasm collections are indispensable for elucidating breeding potential, detecting promising genotypes and breeding climate-resilient cultivars [13]. Phenotypic identification technology, due to its intuitiveness and operability, remains a widely used and practical approach for germplasm evaluation and is complementary to genomic characterization [14].

Significant progress has been made in foxtail millet research through cross-regional germplasm introduction, phenomics evaluation, optimization of cultivation models, and molecular marker-assisted breeding. Based on quantitative descriptions of physicochemical traits and digital image analysis, studies have investigated 25 phenotypic traits including 11 quantitative and 14 qualitative traits, with coefficients of variation ranging from 3.90% to 28.38% for quantitative traits and 7.44% to 66.20% for qualitative traits [15]. Using a recombinant inbred line population, QTL mapping was conducted for nine panicle structure and yield-related traits across diverse environmental conditions, revealing significant variation in traits such as panicle weight, grain weight per panicle, panicle length, panicle diameter and neck protrusion length across different geographical environments [16].

Liu et al. [17] integrated a recombinant inbred line population derived from “Yu Gu No. 1” and “Long Gu No. 7” with whole-genome resequencing data to construct a high-density genetic linkage map comprising 2297 bin markers and 74 SSR markers, and identified identifying 221 QTLs controlling associated with 17 morphological, agronomic and yield-related traits. Upadhyaya et al. [18] performed hierarchical clustering based on phenotypic distance, dividing germplasm into 25 clusters and selecting a mini-core collection comprising 35 accessions representing approximately 10% of the germplasm. However, research on Xinjiang foxtail millet germplasm has several limitations, including sample size constraints (*n* < 100) leading to insufficient germplasm representativeness, heterogeneous evaluation index systems reducing data comparability, and unbalanced trait analysis with excessive focus on panicle characteristics [19]. Particularly for germplasm with typical arid-zone ecotypes in Xinjiang, the existing evaluation system exhibits significant regional adaptability deficiencies [20,21]. Recent advances in foxtail millet genomics have identified numerous QTLs and candidate genes associated with drought tolerance and yield-related traits [17,22]. Our phenotypic characterization provides a foundation for integrating these molecular findings into breeding programs for Xinjiang-adapted germplasm.

This study addresses these knowledge gaps by conducting a systematic phenotypic evaluation of 203 foxtail millet germplasm accessions evaluated in foxtail millet-producing areas of northern Xinjiang. We performed comprehensive phenotypic characterization throughout the entire growth period and analyzed genotype × year variation among morphological characteristics, panicle development, and yield components. We hypothesized that the collection would show substantial phenotypic variation among accessions that is repeatable across the two seasons, and that the accessions could be arranged into phenotypically distinct groups with contrasting yield, architecture and phenology profiles. The specific objectives were to: (1) evaluate phenotypic diversity and phenotypic variation across qualitative and quantitative traits; (2) analyze trait correlations to identify traits associated with yield; (3) conduct principal component analysis to identify major dimensions of phenotypic variation; and (4) perform cluster analysis to classify germplasm into phenotypically distinct groups for candidate breeding applications. The expected outcomes will provide theoretical support for establishing a location-specific phenotype database and for identifying candidate germplasm for future drought-resistant and water-saving evaluation [12,23,24].

## 2. Materials and Methods

### 2.1. Experimental Materials

This study evaluated 203 foxtail millet germplasm accessions collected from major foxtail millet-producing regions of China. The materials comprised, 31 landraces, 50 cultivars and 122 breeding lines from 15 provinces and regions of China, mainly in, including Shanxi (54 accessions), Xinjiang (35), Inner Mongolia, Shanxi, Hebei, Gansu, and other provinces (23) and Hebei (20) (Supplementary Table S1). Accessions were selected to capture the breadth of phenotypic diversity present in Chinese foxtail millet germplasm, with particular emphasis on materials adapted to Xinjiang’s arid and semi-arid conditions.

### 2.2. Experimental Design

The field trial was conducted over two consecutive growing seasons (2023 and 2024) at Xisi Village, Kan’erzi Township, Qitai County, Xinjiang Uygur Autonomous Region (44.02° N, 89.57° E). This location is characterized by a typical continental climate with significant temperature fluctuations, limited precipitation, and relatively short frost-free periods [25,26]. The previous crops were sorghum in 2023 and buckwheat in 2024. The experiment employed a randomized complete block design with three replicate blocks in each year. Each experimental plot measured 8 m^2^ (5 m × 1.6 m) and was sown on 1 May 2023 and 5 May 2024 (Supplementary Table S3A) using a locally adapted plastic film mulching system with hole-seeding at 40 cm row spacing and 10 cm hill spacing. The fields were prepared on 18 April 2023 and 25 April 2024, and diammonium phosphate (150 kg·ha^−1^) was applied as basal fertilizer on 16 April in both years (Supplementary Table S3A). The seeding rate was approximately 8.4 kg·ha^−1^, with approximately 3–4 seeds sown per hill to achieve the target plant density of approximately 250,000 plants ha^−1^ at maturity. All plots received uniform irrigation six times during the growth cycle, at emergence, jointing (two irrigations), heading, and grain filling (two irrigations), with a total seasonal irrigation volume of 5550 m^3^·ha^−1^ (555 mm) in both years, applied in monthly amounts of 750–1200 m^3^·ha^−1^ (specific dates in Supplementary Table S3A). Plots were harvested on 5 September 2023 and 10 September 2024 (Supplementary Table S3A). Standardized field management practices were maintained throughout, and border rows surrounded the experimental area to minimize edge effects.

### 2.3. Trait Evaluation Methods

#### 2.3.1. Qualitative Trait Assessment

Eight qualitative traits were visually evaluated following the descriptors and data standards for foxtail millet germplasm resources [5]. The traits assessed included: seedling leaf color (SLC), leaf sheath color (LSC), seedling leaf posture (SLP), leaf posture during flowering (LPDF), panicle neck shape (NS), panicle pattern (TP), panicle elasticity (SE), and grain color (GC) (Table 1). Ten plants per plot were scored individually; the modal category per plot was used for frequency analysis, and plot-level modal values were used as the unit of analysis.

#### 2.3.2. Quantitative Trait Measurement

Thirteen quantitative traits were assessed according to standard descriptors [12,27], with ten randomly selected plants per plot evaluated for each trait. The growth period (GP, days) was defined as the interval from seedling emergence to physiological maturity. Plant height (PH, cm) was recorded as the distance from the soil surface to the apex of the main panicle. Main stem diameter (MSD, mm) was determined at the first internode above the soil surface using digital calipers. The number of main stem nodes (NMSN) was counted on the main culm at maturity. Leaf length (LL, cm) was measured from the ligule to the tip of the flag leaf, while leaf width (LW, cm) was recorded at the widest point of the flag leaf blade using digital calipers. Panicle length (EL, cm) was measured from the basal node of the main panicle to its apex, and panicle diameter (ED, mm) was recorded at the widest point of the main panicle.

Thousand grain weight (TGW, g) was obtained by counting 1000 clean, air-dried grains and weighing them on a precision balance. Single panicle weight (SEW, g) was defined as the total weight of the intact main panicle including grains and rachis, measured after air-drying. Grain weight per panicle (GWE, g) was defined as the weight of threshed grains obtained from the same main panicle. All panicles were air-dried to constant moisture before weighing, and moisture content was not further adjusted for these two traits.

### 2.4. Data Analysis

#### 2.4.1. Phenotypic Diversity Analysis

Phenotypic diversity of qualitative traits was assessed using the Shannon–Weaver diversity index [28]: H′ = −Σ (p_i_ × ln p_i_), where p_i_ represents the phenotypic frequency of category i [29]. For quantitative traits, each trait was divided into 10 classes using the two-year accession means: class 1 contained values below the mean − 2 SD, classes 2–9 were successive intervals of 0.5 SD (lower boundary included, upper boundary excluded) and class 10 contained values of at least the mean + 2 SD; H′ was calculated from the class frequencies (maximum H′ = ln 10 = 2.303). Quantitative trait variation was evaluated using the coefficient of variation (CV = standard deviation/mean × 100%) [30].

#### 2.4.2. Correlation and Multivariate Statistical Analysis

All descriptive, correlation, PCA, cluster and regression analyses used the two-year mean of each accession (n = 203). Pearson correlation analysis was performed to examine relationships among quantitative traits using Origin 2024 software. Principal component analysis (PCA) was conducted on the correlation matrix of the 12 traits remaining after excluding SEW (i.e., all variables were standardized to zero mean and unit variance), using SPSS software (Version 26.0, IBM Corp., Armonk, NY, USA) to identify the major axes of phenotypic variation and reduce the dimensionality of the trait dataset [31]. Components with eigenvalues above 1 were retained and unrotated loadings were reported; loadings of 0.40 or more in absolute value were considered high. Hierarchical cluster analysis was performed using the unweighted pair group method with arithmetic mean (UPGMA) based on squared Euclidean distance [32], computed on the unstandardized means of the same 12 traits (yield in kg·mu^−1^). The dendrogram was cut where it gave four groups; the rationale is given in Section 3.8. Differences among groups were described by one-way ANOVA, which is descriptive because the groups were formed from the same traits.

#### 2.4.3. Combined Analysis of Variance and Repeatability

Combined analysis of variance (ANOVA) was performed on the two-year dataset using one mean per accession per year, with accession (genotype, G) and year (E) as factors. Because there was a single observation per accession per year, the G × Y interaction could not be separated from residual error, and its mean square (df = 202) was used as the error term for testing G and E [33]. Broad-sense heritability cannot be estimated from this design, so entry-mean repeatability (R) was calculated instead: R = 1 − MS(G × Y)/MS(G), which equals σ^2^G/(σ^2^G + σ^2^GY/e) with e = 2 years. R describes how consistently accession means were reproduced across the two seasons at this site. Multiple comparisons among genotype means were performed using the LSD method (*p* < 0.05). The complete dataset of genotype means for all traits across both evaluation years is presented in Supplementary Table S2.

#### 2.4.4. Regression Analysis

Multiple regression analysis was conducted using SPSS 26.0 to examine associations between grain yield and quantitative traits. Because single panicle weight and grain weight per panicle are components of the same harvested panicle, their joint inclusion may introduce mathematical redundancy; this is acknowledged in the Discussion. Model assumptions were checked using residual diagnostics, leverage and influence statistics, and variance inflation factors. Predictive performance was evaluated using cross-validation. Stepwise selection was used descriptively to explore associations and not to infer causal importance.

## 3. Results

### 3.1. Phenotypic Diversity of Qualitative Traits

The eight qualitative traits of the 203 foxtail millet accessions exhibited varying levels of phenotypic diversity (Figure 1). Among the qualitative traits, leaf posture during flowering (LPDF) showed the highest phenotypic diversity index (H′ = 1.031), indicating broad phenotypic variation for this trait. This was followed by seedling leaf posture (SLP, H′ = 0.928), panicle neck shape (NS, H′ = 0.869), spike elasticity (SE, H′ = 0.801), seedling leaf color (SLC, H′ = 0.718), panicle pattern (TP, H′ = 0.652), grain color (GC, H′ = 0.628), and leaf sheath color (LSC, H′ = 0.473). The phenotypic diversity indices ranked from high to low as follows: LPDF > SLP > NS > SE > SLC > TP > GC > LSC. These results indicate substantial phenotypic differences among the traits, reflecting the phenotypic diversity present in the germplasm collection.

### 3.2. Distribution Patterns of Qualitative Traits

The distribution types and frequencies of the eight qualitative traits revealed distinct variation patterns within the germplasm collection (Table 2). At the seedling stage, seedling leaf color was predominantly green (77.83%), followed by purple-green (12.81%) and yellow-green (9.36%). Leaf sheath color was overwhelmingly green (91.13%), with only small proportions exhibiting purple (5.42%) or red (3.45%). Seedling leaf posture was primarily semi-erect (57.14%), followed by horizontal (34.98%), erect (4.43%), and drooping (3.45%).

During the flowering stage, leaf posture was mainly drooping (50.74%), followed by horizontal (35.96%), semi-erect (6.90%), and erect (6.40%). The panicle neck shape of most accessions was curved (74.88%), with moderately curved (8.87%), hook-shaped (8.87%), and straight (7.39%) types constituting the remainder. At maturity, panicle pattern was predominantly spindle-shaped (83.25%), followed by cylindrical (9.85%), club-shaped (6.40%), and cat-claw-shaped (0.49%). Panicle elasticity was primarily intermediate (76.35%), with compact (15.27%) and loose (8.37%) types representing the remaining proportion. Grain color was mostly yellow (84.73%), with white (11.33%), brown (1.97%), red (1.48%), and black (0.49%) grains present; orange and cyan grain colors were absent from this collection. These distribution patterns provide valuable baseline information for germplasm management and targeted breeding strategies.

### 3.3. Phenotypic Variation and Diversity of Quantitative Traits

Analysis of the 13 quantitative traits revealed considerable variation in both coefficient of variation (CV) and Shannon–Weaver diversity index (H′) across the 203 accessions (Table 3). Grain weight per panicle (GWE) exhibited the highest coefficient of variation (38.91%), followed by single panicle weight (SEW, 37.65%), yield (Y, 37.31%), dry matter accumulation per plant (DMA, 27.13%), panicle length (EL, 24.72%), panicle diameter (ED, 20.35%), leaf length (LL, 17.16%), leaf width (LW, 13.73%), main stem diameter (MSD, 11.72%), number of main stem nodes (NMSN, 11.20%), thousand grain weight (TGW, 9.24%), growth period (GP, 8.57%), and plant height (PH, 7.32%). The high CV values for GWE, Y and SEW indicate substantial phenotypic variation and potential for selection for yield improvement. As expected, the mean GWE (12.18 g) was lower than the mean SEW (16.85 g). The ranges and CVs of EL (11.25–78.24 cm) and LL (29.33–113.16 cm) are inflated by a few extreme values that are unusually large for foxtail millet: the panicle length of Jiugu 19 (No. 131; 77.84 cm in 2023 and 78.64 cm in 2024) and the leaf length of Youza 2 (No. 94; 112.69 cm in 2023 and 113.64 cm in 2024). These values were checked against the original data sheets; they were recorded in both years and were therefore retained in all analyses. Excluding these two accessions would lower the CV of EL from 24.72% to 17.07% and that of LL from 17.16% to 11.79%. Because these traits are measured on the same panicle or plot, their variation may include both genotypic and environmental components.

Shannon–Weaver diversity indices, calculated with 10 classes (maximum H′ = 2.303), ranged from 1.697 (LL) to 2.090 (DMA). H′ was highest for dry matter accumulation (2.090), followed by main stem diameter (2.079), leaf width (2.075), thousand grain weight (2.066), yield (2.066), growth period (2.049), plant height (2.039), grain weight per panicle (2.027), number of main stem nodes (2.022), single panicle weight (2.000), panicle diameter (1.974), panicle length (1.736) and leaf length (1.697). Most traits therefore had similar H′ values (1.97–2.09); panicle length and leaf length were lower because a few extreme values widened the class intervals and concentrated the remaining accessions in the central classes. Because H′ of a continuous trait depends on the class scheme and is almost constant for approximately normal traits, the CV is the more informative measure of relative variability among these traits.

### 3.4. Frequency Distribution of Quantitative Traits

The frequency distribution analysis of the 13 quantitative traits revealed distinct distribution patterns and variation characteristics among the tested materials (Figure 2). Most traits were approximately normally distributed (Shapiro–Wilk *p* > 0.05 for PH, MSD, LW, SEW, GWE, TGW, DMA and Y), whereas GP, NMSN and ED deviated slightly from normality and EL and LL were strongly right-skewed (skewness 5.6 and 5.7) because of a few extreme values. Growth period showed a mildly right-skewed distribution, with 64.5% of accessions in the range of 95–110 days. Grain weight per panicle was approximately symmetric, with 51.7% of accessions between 10 and 16 g, consistent with its high CV (38.88%) and indicating large phenotypic heterogeneity among accessions.

Thousand grain weight was concentrated in the range of 2.40–2.80 g (63.5% of accessions), with a few extreme values. Yield showed a wide distribution, with the most frequent class at 3750–4500 kg·ha^−1^ (250–300 kg·mu^−1^; 22.7% of accessions), consistent with its relatively high CV (37.31%) and indicating its importance as a selection target. For plant morphology-related traits, most accessions had a plant height of 160–200 cm (86.2%), 10–14 main stem nodes (69.0%), a main stem diameter of 6.0–8.5 mm (80.3%), a leaf length of 35–45 cm (75.4%) and a leaf width of 1.8–2.8 cm (90.1%). Although NMSN and LW showed tendencies toward concentration, they still maintained moderate levels of variation, with CV values of 11.46% and 13.45%, respectively, and moderate potential for selection. Panicle diameter was approximately symmetric, whereas panicle length was strongly right-skewed because of extreme values. Panicle length was concentrated in the range of 18–26 cm (72.9% of accessions), and panicle diameter was concentrated in the range of 16–28 mm (84.7%). These were consistent with their moderate CVs (24.72% and 18.77%, respectively), indicating potential for selection.

### 3.5. Correlation Analysis Based on Quantitative Traits

Correlation analysis revealed complex relationships among the 13 quantitative traits (Figure 3). Yield showed a highly significant positive correlation with both single panicle weight (SEW, r = 0.925, *p* < 0.001) and grain weight per panicle (GWE, r = 0.925, *p* < 0.001), and SEW and GWE were almost collinear (r = 0.996, *p* < 0.001). This indicates that panicle weight and grain weight per panicle are closely associated with yield [14]; because these traits are measured on the same panicles and plots, the association partly reflects measurement dependence rather than independent effects.

Thousand grain weight (TGW) was not significantly correlated with yield (r = 0.050, *p* = 0.47) or with grain weight per panicle (r = 0.055, *p* = 0.44), suggesting that in this collection differences in yield and grain weight per panicle were not associated with grain size [34]. Plant height showed significant positive correlations with number of main stem nodes (r = 0.344, *p* < 0.001) and main stem diameter (r = 0.261, *p* < 0.001), and main stem diameter and node number were themselves positively correlated (r = 0.570, *p* < 0.001); taller accessions therefore tended to have more nodes and thicker stems (lodging was not measured) [35]. Dry matter accumulation was positively correlated with number of main stem nodes (r = 0.468, *p* < 0.001), main stem diameter (r = 0.441, *p* < 0.001), GWE (r = 0.405, *p* < 0.001), SEW (r = 0.400, *p* < 0.001), yield (r = 0.341, *p* < 0.001), plant height (r = 0.279, *p* < 0.001) and panicle diameter (r = 0.278, *p* < 0.001) but not with panicle length (r = −0.049, *p* = 0.49). Panicle diameter was also positively correlated with main stem diameter (r = 0.333, *p* < 0.001) and node number (*r =* 0.312, *p* < 0.001) and weakly with GWE (*r* = 0.242, *p* < 0.001), whereas panicle length and panicle diameter were not significantly correlated (*r* = 0.083, *p* = 0.24) [34]. Growth period was not significantly correlated with any other trait (|*r*| ≤ 0.033). Leaf length was not significantly correlated with leaf width (*r* = 0.037, *p* = 0.61), and leaf width showed only weak positive correlations with SEW (*r* = 0.169, *p* = 0.016), GWE (*r* = 0.153, *p* = 0.029) and yield (*r* = 0.152, *p* = 0.031); these weak associations do not demonstrate an effect of leaf area on sink capacity [36].

### 3.6. Stepwise Regression Analysis

Stepwise multiple regression analysis (yield in kg·ha^−1^ as the dependent variable; the 11 traits other than yield and SEW as candidate predictors) retained two significant predictors: grain weight per panicle (GWE) and main stem diameter (MSD). The regression equation was:

Yield = 1161.30 + 286.73(GWE) − 110.43(MSD)

The model explained 86.1% of the variation in yield (R^2^ = 0.861, adjusted R^2^ = 0.859, *p* < 0.001; 10-fold cross-validated *R*^2^ = 0.855, RMSE = 502 kg·ha^−1^). Standardized regression coefficients showed that GWE was by far the strongest predictor (β = 0.938), whereas MSD contributed little (β = −0.078). The variance inflation factors were 1.03 for both predictors, residuals did not depart from normality (Shapiro–Wilk *p* = 0.17), and no observation was unduly influential (maximum Cook’s distance = 0.054). TGW and DMA were not retained. Adding SEW raised R^2^ only to 0.865 and made the model collinear (VIF ≈ 50), which is why SEW was excluded.

### 3.7. Principal Component Analysis Based on Quantitative Traits

Principal component analysis was conducted on 12 standardized quantitative traits (SEW excluded to avoid redundancy with GWE). Five principal components had eigenvalues above 1 (2.837, 1.823, 1.180, 1.024 and 1.002), cumulatively accounting for 65.548% of the total phenotypic variation (Table 4); PC5 only just exceeded the threshold. The first five components therefore summarized about two-thirds of the phenotypic variation among the accessions.

Principal component 1 (PC1) had a contribution rate of 23.645% and was labelled the “biomass–productivity factor.” It showed high positive loadings for dry matter accumulation (0.770), main stem diameter (0.677), grain weight per panicle (0.657), number of main stem nodes (0.632), yield (0.593) and panicle diameter (0.562). Principal component 2 (PC2) had a contribution rate of 15.188% and was labelled the “yield–stature contrast.” Yield (0.741) and grain weight per panicle (0.694) loaded positively and number of main stem nodes (−0.503), plant height (−0.468) and main stem diameter (−0.417) loaded negatively. Principal component 3 (PC3) had a contribution rate of 9.832% and was labelled the “panicle length and grain size factor,” with high loadings for panicle length (0.757), thousand grain weight (0.490) and leaf length (0.413). Principal component 4 (PC4) had a contribution rate of 8.532% and was labelled the “phenology and leaf factor,” with high loadings for growth period (0.576) and leaf length (0.474). Principal component 5 (PC5) had a contribution rate of 8.351% and was labelled the “grain size and leaf width factor,” with high loadings for thousand grain weight (0.647) and leaf width (0.425). These labels are descriptive; photosynthesis, lodging and grain number were not measured.

### 3.8. Cluster Analysis Based on Quantitative Traits

Hierarchical cluster analysis using the UPGMA method and squared Euclidean distance, based on the unstandardized two-year means of 12 quantitative traits (excluding SEW to avoid redundancy; yield in kg·mu^−1^), classified the 203 accessions into four groups when the dendrogram was cut between the fusion distances of 7790 and 12,382 (Figure 4; Table 5). Because the traits were not standardized, yield accounted for 94.7% of the total variance and largely determined the grouping, so the groups mainly reflect yield level and the panicle traits associated with it. Four groups were retained because the fusion distance rose sharply beyond this level (12,382 compared with 7790 at the previous step) and because the groups differed significantly in yield and panicle weights (Table 5).

Group I comprised 129 accessions (63.55% of the collection), the largest cluster. It was intermediate for yield (3532 ± 670 kg·ha^−1^), SEW (16.88 g) and GWE (11.30 g), and its trait means were close to the collection means (Table 5). Its passport composition (60.5% breeding lines, 21.7% cultivars and 17.8% landraces) resembled that of the whole collection, and it included accessions from many provinces (for example, Shanxi 32, Xinjiang 19 and Shaanxi 14), so it cannot be described as predominantly landraces. Group II included 37 accessions (18.23%) with the lowest yield (1650 ± 414 kg·ha^−1^), SEW (8.37 g), GWE (4.85 g) and dry matter accumulation (21.66 g) (all *p* < 0.001); its mean plant height (182.72 cm) and main stem diameter (7.05 mm) were similar to those of Groups I and III. Group III included 32 accessions (15.76%) with high yield (5282 ± 460 kg·ha^−1^), SEW (23.90 g), GWE (16.21 g) and dry matter accumulation (29.88 g). Its members came from Shanxi (9), Inner Mongolia (6), Xinjiang (4) and other provinces and included 12 cultivars. Group IV contained only 5 accessions (2.46%), all breeding lines (four from Inner Mongolia and one from Xinjiang), with the highest yield (6814 ± 381 kg·ha^−1^), SEW (29.57 g), GWE (21.13 g), dry matter accumulation (33.35 g) and main stem diameter (8.39 mm). Their mean growth period (104.1 d; range 99.2–107.0 d) was 2.6 days shorter than the collection mean, but growth period did not differ significantly among the groups (*p* = 0.75). The groups also did not differ significantly in plant height, panicle length, leaf length, leaf width, number of main stem nodes or thousand grain weight (all *p* > 0.05) and differed only weakly in panicle diameter (*p* = 0.042). Because the groups were formed from the same traits, these tests are descriptive. Germplasm status did not differ significantly among the groups (χ^2^ test, *p* = 0.27).

### 3.9. Analysis of Variance and Repeatability

Combined analysis of variance across the two growing seasons (2023 and 2024) revealed highly significant effects of genotype (G) for all 13 traits evaluated (*p* < 0.001), indicating substantial phenotypic variation among accessions (Table 6). The accession means for each trait and year are presented in Supplementary Table S2. The environmental main effect (year, E) was significant for all traits (*p* < 0.01 or *p* < 0.001), reflecting year-to-year differences at the experimental site (climatic drivers were not analysed); the 2024 means exceeded the 2023 means for all 13 traits. The genotype × year (G × Y) mean square, which also contains residual error, was between 0.01% and 28% of the genotype mean square (Table 6), indicating that accession performance was repeatable across the two seasons at this site.

Entry-mean repeatability (R = 1 − MS(G × Y)/MS(G); Section 2.4.3) was very high for yield (99.99%), dry matter accumulation (99.9%), grain weight per panicle (99.9%), panicle length (99.7%), panicle diameter (99.7%), single panicle weight (99.6%), leaf length (99.6%), number of main stem nodes (99.0%), plant height (98.9%) and main stem diameter (97.6%), high for growth period (94.9%), and moderate to high for leaf width (79.1%) and thousand grain weight (72.1%). These estimates indicate good year-to-year repeatability of accession means at this site. They are likely upper bounds, because G × Y and residual error could not be separated, and they may not apply to other locations or to water-limited conditions. For yield, the correlation between the two seasons’ accession means was 0.9997.

## 4. Discussion

### 4.1. Phenotypic Diversity Patterns in Foxtail Millet Germplasm

Principal component analysis of 12 standardized traits yielded five components with eigenvalues above 1, which cumulatively accounted for 65.548% of the variance. These components can be described as a biomass–productivity factor (dry matter accumulation, stem diameter, grain weight per panicle, node number, yield and panicle diameter), a yield–stature contrast (yield and grain weight per panicle against node number, plant height and stem diameter), a panicle length and grain size factor (panicle length, thousand grain weight and leaf length), a phenology–leaf factor (growth period and leaf length) and a grain size–leaf width factor (thousand grain weight and leaf width). Rao and Chaturvedi [37] reported that the first five principal components cumulatively explained 82.43% of the variation, with the first principal component being a yield factor (explaining 37.50%), followed by plant morphology, ear characteristics and growth stage factors. Our first components are similarly dominated by yield-related and plant-size traits, but our five components explain less of the variance, which reflects the weak correlations among most of the remaining traits (for example, growth period was not correlated with any other trait). This indicates that, despite differences in germplasm populations and traits examined, yield potential and plant architecture are recurrent dimensions of phenotypic variation in foxtail millet.

In Table 4, high PC1 loadings were found for dry matter accumulation (0.770), main stem diameter (0.677), grain weight per panicle (0.657), number of main stem nodes (0.632), yield (0.593) and panicle diameter (0.562), whereas growth period (0.016), thousand grain weight (−0.041) and panicle length (0.167) did not load on PC1. Yield and grain weight per panicle loaded on PC2 (0.741 and 0.694) with signs opposite to those of number of main stem nodes (−0.503), plant height (−0.468) and main stem diameter (−0.417), so on this axis accessions with higher grain yield tended to be shorter and to have fewer nodes and thinner stems. Because lodging and stem reserves were not measured, the PCA does not by itself show a causal trade-off or a stem storage function [38].

### 4.2. Phenotypic Grouping and Breeding Implications

Phenotype-based cluster analysis divided the 203 accessions into four groups that differed significantly in yield, panicle weights and dry matter accumulation but not in growth period or in most plant-architecture traits. Passport data (Supplementary Table S1) show that the groups did not differ significantly in germplasm status (*p* = 0.27) and were not clearly associated with province of origin, except that four of the five Group IV accessions came from Inner Mongolia.

Group I (129 accessions, 63.55%) contained most of the collection and was intermediate for yield and panicle traits. Because it is simply the largest cluster, it should not be regarded as a core set: a core collection requires dedicated selection (for example, stratified or maximization-based sampling) and validation of how well it represents the variation in the whole collection, which was not done here. Group II (37 accessions, 18.23%) had the lowest yield (1650 kg·ha^−1^) and panicle weights, and its plant height and stem diameter were similar to those of the other groups, so these accessions cannot be proposed as lodging-resistance candidates; lodging was not scored. Group III (32 accessions, 15.76%) had high yield (5282 kg·ha^−1^) and grain weight per panicle (16.21 g). These materials are candidates for high-yield breeding; their value as parents would require validation in multi-location trials and cannot be inferred from this single irrigated site. Group IV (5 accessions, 2.46%) consisted of breeding lines (four from Inner Mongolia) with the highest yield (6814 kg·ha^−1^), grain weight per panicle (21.13 g) and stem diameter (8.39 mm) and a growth period only slightly shorter than average (104.1 d), so it is a small candidate pool for yield improvement rather than an early-maturing group. The yields of Groups III and IV are not directly comparable with those reported by Zhao et al. [12] for 197 germplasm resources from the Inner Mongolia region because environments and management differed. Growth period, which matters for short-season systems [39], did not differ significantly among the groups (*p* = 0.75). The groups separate mainly along yield and panicle-weight axes. As hypotheses for future testing, crosses between Group III or IV accessions and accessions carrying other desirable traits (for example, early maturity or lodging resistance, which would first have to be identified) could be evaluated; such crosses have not been tested.

### 4.3. Genotype × Year Variation and Repeatability

Combined analysis of variance across the two growing seasons (2023 and 2024) showed highly significant genotype (G) effects for all 13 traits analysed, indicating considerable phenotypic variation within the collection. The year effect was significant for all traits, reflecting inter-annual differences at the experimental site. The G × Y mean square, which also contains residual error, was small relative to the genotype mean square (Table 6). Because only one site was evaluated, this indicates year-to-year repeatability of accession means at this location rather than broad environmental stability.

Entry-mean repeatability was very high for yield (99.99%), dry matter accumulation (99.9%), grain weight per panicle (99.9%), single panicle weight (99.6%), number of main stem nodes (99.0%), plant height (98.9%) and main stem diameter (97.6%), high for growth period (94.9%) and panicle diameter (91.4%), and moderate to high for panicle length (80.2%), leaf width (79.1%), leaf length (74.0%) and thousand grain weight (72.1%). Such values are likely to be higher than heritability estimated from a full plot-level mixed model, because replicate error and G × Y interaction could not be separated and only two seasons at one site were evaluated; they should be re-estimated with variance components (σ^2^G, σ^2^GY, σ^2^e) and confidence intervals. The trial was conducted under irrigated conditions with six uniform irrigations during the growth cycle; consequently, these estimates may not be directly transferable to dryland or rainfed environments, where water stress could intensify G × E interaction and reduce the proportion of variance attributable to genotype [23,40]. Future multi-location, multi-environment trials that include dryland settings are recommended.

### 4.4. Comparison with Related Studies

The phenotypic diversity indices observed in this study are generally comparable to or higher than those reported in other foxtail millet germplasm evaluations. Xue [15] reported Shannon diversity indices ranging from 0.410 to 0.809 for 1558 foxtail millet germplasm resources, while our study found H′ values up to 2.107 for quantitative traits (10-class scheme). H′ values are not directly comparable between studies because they depend on the traits and class definitions used. The mean yields of Group III (5282 kg·ha^−1^) and Group IV (6814 kg·ha^−1^) are higher than the yields reported in some previous studies. For example, Zhao et al. [12] reported yield means of approximately 4500–5000 kg·ha^−1^ for foxtail millet germplasm in Inner Mongolia. Yields from different trials (irrigated and plastic-film mulched here) are not directly comparable, so this difference should not be interpreted as superior genetic performance without multi-location testing.

The correlation patterns observed between yield and yield components are consistent with previous findings in foxtail millet. Liu et al. [14] found that grain weight per panicle and single panicle weight were strongly associated with yield in foxtail millet. These findings are consistent with the importance of panicle productivity for grain yield in millet species, although in this dataset the traits share a common harvest and SEW and GWE are almost collinear (r = 0.99), so their separate contributions to yield cannot be distinguished.

### 4.5. Limitations

This study has several limitations. Only one site was evaluated over two seasons, so genotype × environment effects and broad adaptation across northern Xinjiang could not be assessed. The trial was fully irrigated, so drought tolerance and water-saving performance were not tested. Lodging, harvest index, forage quality, disease response and grain quality were not measured, so claims about these attributes remain hypotheses. Diversity, clustering and grouping are based on phenotypes only; no molecular or pedigree data were used, so they should not be equated with genetic diversity or population structure. The ANOVA used one accession mean per year, so G × Y could not be separated from error and the repeatability estimates are upper bounds; variance components should be re-estimated from plot-level data. The cluster analysis used unstandardized values and is dominated by yield, and the extreme panicle-length and leaf-length values noted in Section 3.3 still require verification against the original records. Group I should not be treated as a core collection without formal evaluation.

### 4.6. Future Perspectives

While this study provides comprehensive phenotypic characterization, future research should integrate these phenotypic evaluation results with molecular marker genotyping analysis to conduct genome-wide association studies (GWASs) or allele mining, elucidating the genetic basis of important agronomic traits [22,41]. The distinctive groups identified in this study can be utilized for targeted hybridization, and the combined application of phenotypic and genotypic information can accelerate the genetic improvement and innovation of foxtail millet varieties [17,34]. Additionally, the high-yielding groups (Groups III and IV) are candidate resources whose value in addressing production challenges remains to be validated. Future research should focus on: (i) conducting multi-environment trials under both irrigated and dryland conditions; (ii) integrating phenotypic and genotypic data for GWAS analysis; (iii) developing molecular markers for key yield and stress tolerance traits; (iv) utilizing the distinctive groups for targeted hybridization and selection; and (v) assessing the nutritional quality and processing characteristics of superior genotypes.

## 5. Conclusions

This study evaluated 203 foxtail millet accessions for 8 qualitative and 13 quantitative traits over two seasons at a single irrigated site in northern Xinjiang. The collection showed substantial phenotypic diversity: among qualitative traits, leaf posture during flowering had the highest H′ (1.072), and among quantitative traits, grain weight per panicle (CV = 38.88%), yield (37.31%) and single panicle weight (35.21%) were the most variable. Yield was strongly associated with single panicle weight and grain weight per panicle (r = 0.925 for both), although these traits come from the same harvest and partly overlap (SEW and GWE: r = 0.990). Principal component analysis (five components, 65.55% of variance) and cluster analysis separated four phenotypic groups that differed mainly in yield and panicle weight: Group IV (5 accessions) and Group III (32) had the highest yields, Group I (129) had an intermediate yield and Group II (37) had the lowest yield. Groups III and IV are candidates for follow-up work in high-yield breeding. Lodging, drought response, forage value and stability across locations were not measured, so these uses require validation, and the repeatability estimates, which are upper bounds, should be re-estimated from plot-level data. Integrating molecular data and multi-location trials, including water-limited conditions, is the logical next step.

## Figures and Tables

**Figure 1:**
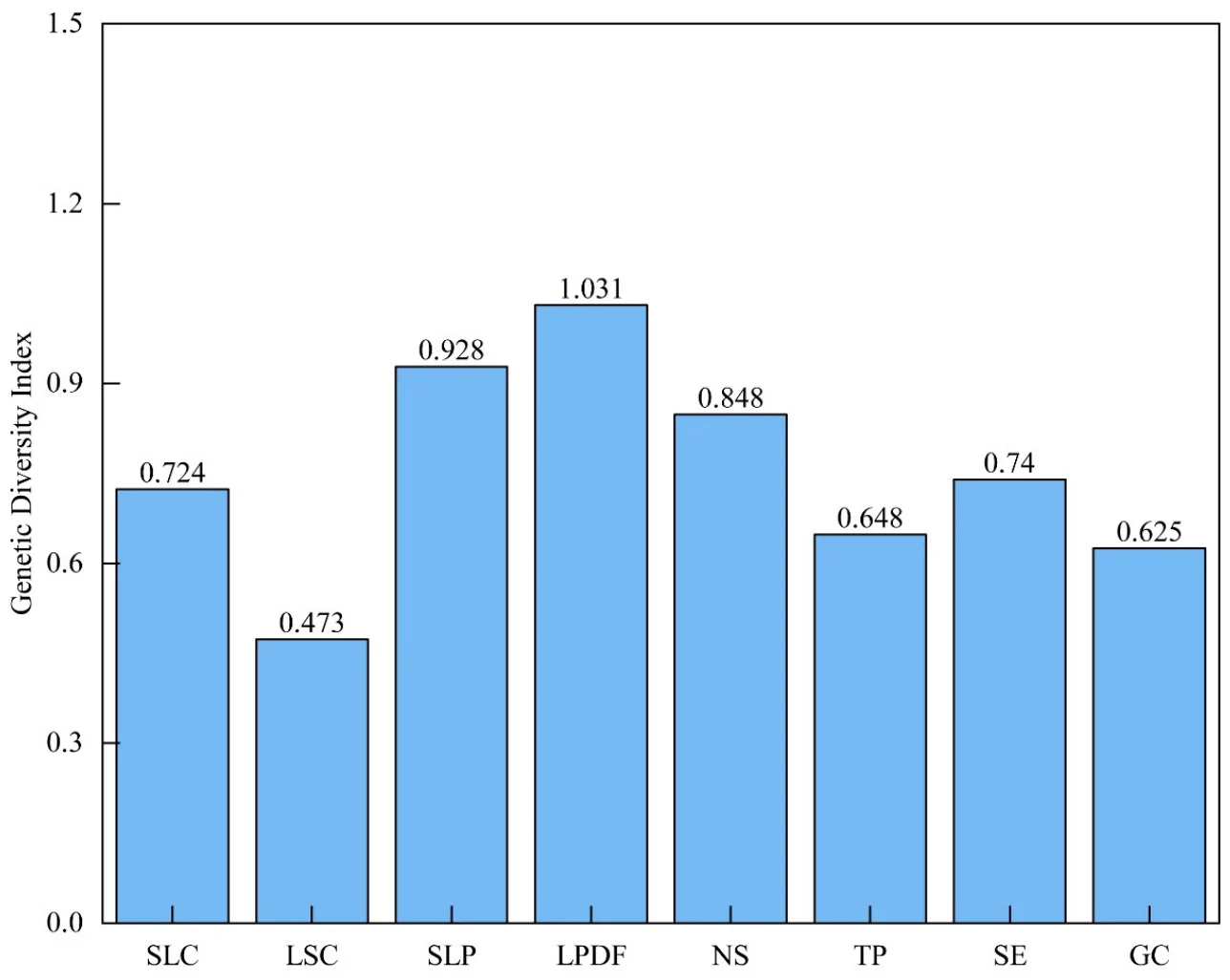
Phenotypic diversity indices (H′) of eight qualitative traits in 203 foxtail millet germplasm accessions. Traits: SLC, seedling leaf color; LSC, leaf sheath color; SLP, seedling leaf posture; LPDF, leaf posture during flowering; NS, panicle neck shape; TP, panicle pattern; SE, panicle elasticity; GC, grain color.

**Figure 2:**
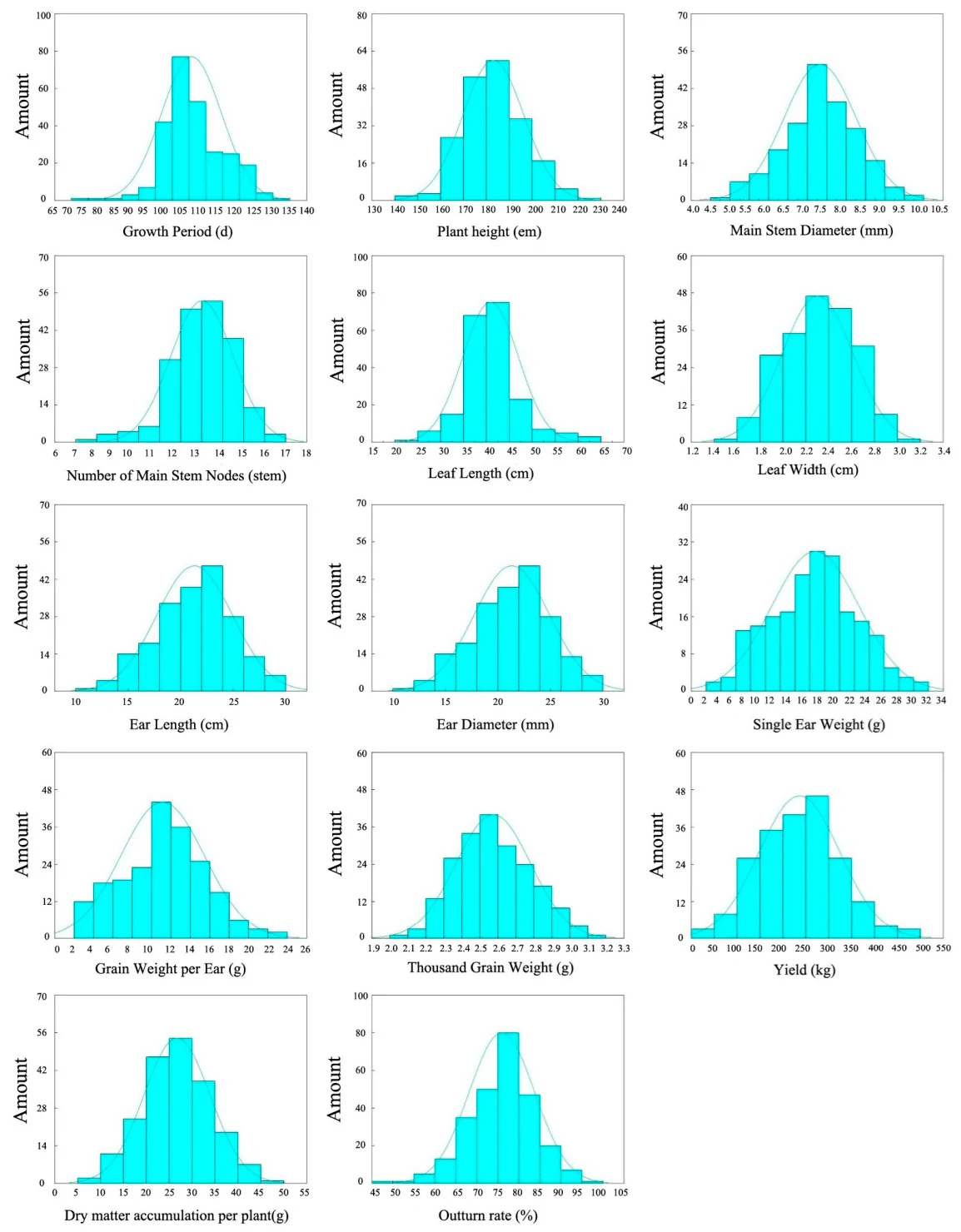
Frequency distribution of 13 quantitative traits in 203 foxtail millet germplasm accessions, growth period; plant height; main stem diameter; number of main stem nodes; leaf length; leaf width; panicle length, panicle diameter, thousand grain weight; single panicle weight, grain weight per panicle, dry matter accumulation, yield.

**Figure 3:**
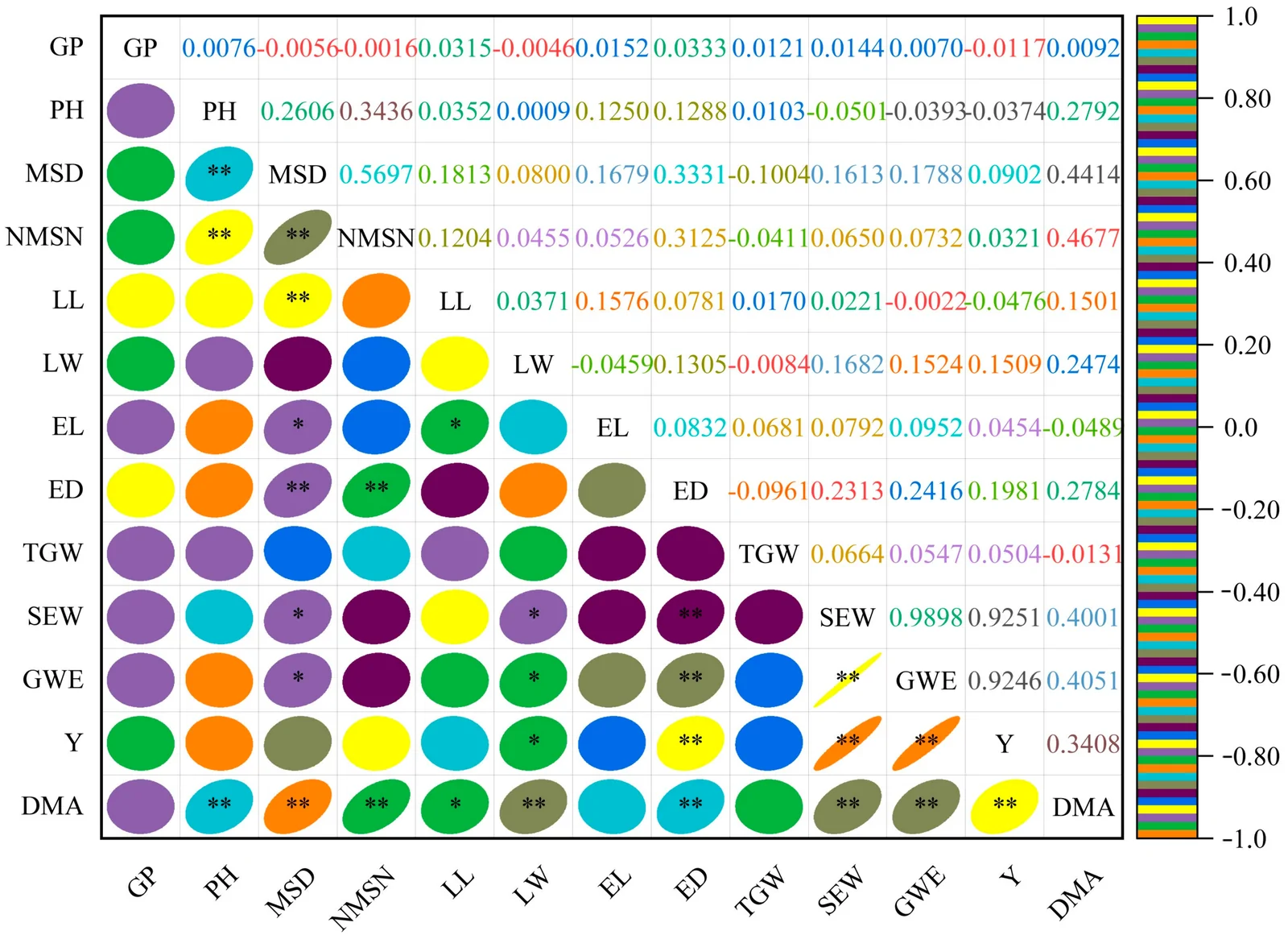
Correlation matrix of 13 quantitative traits in 203 foxtail millet germplasm accessions. GP, growth period; PH, plant height; MSD, main stem diameter; NMSN, number of main stem nodes; LL, leaf length; LW, leaf width; EL, panicle length; ED, panicle diameter; TGW, thousand grain weight; SEW, single panicle weight; GWE, grain weight per panicle; DMA, dry matter accumulation; Y, yield. Values represent Pearson correlation coefficients; *, and ** indicate significance at *p* < 0.05, and *p* < 0.01, respectively.

**Figure 4:**
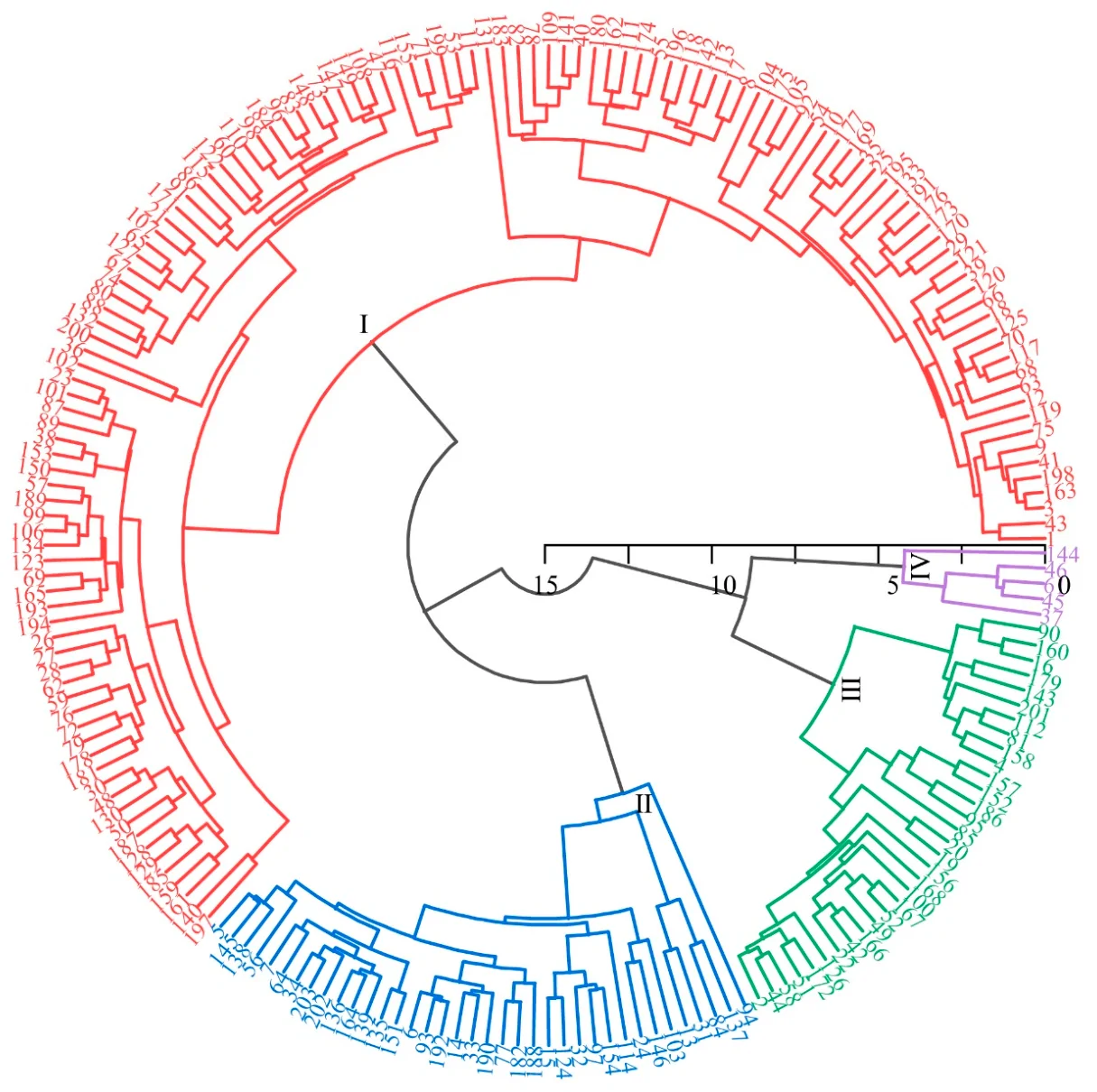
Hierarchical cluster analysis of 203 foxtail millet germplasm accessions based on 12 quantitative traits (excluding single panicle weight) using the UPGMA method with squared Euclidean distance. The four distinct groups (I–IV) are color-coded.

**Table 1:** Description Criteria for Eight Qualitative Traits of Foxtail Millet Germplasm Resources.

Trait	Descriptive Criteria
Seedling leaf color/SLC	1. Green, 2. Yellow-green, 3. Purple-green
Leaf sheath color/LSC	1. Green, 2. Red, 3. Purple
Seedling leaf posture/SLP	1. Erect, 2. Semi-erect, 3. Horizontal, 4. Drooping
Leaf posture during flowering/LPDF	1. Erect, 2. Semi-erect, 3. Horizontal, 4. Drooping
Neck shape/NS	1. Straight, 2. Moderately curved, 3. Curved, 4. Hook-shaped
Panicle pattern/TP	1. Chicken-beak-shaped, 2. Spindle-shaped, 3. Cylindrical, 4. Club-shaped, 5. Duck-bill-shaped, 6. Cat-claw-shaped, 7. Buddha’s-hand-shaped
Panicle elasticity/SE	1. Loose, 2. Intermediate, 3. Compact
Grain color/GC	1. White, 2. Yellow, 3. Orange, 4. Red, 5. Cyan, 6. Brown, 7. Black

**Table 2:** Frequency Distribution of Variation Types for 8 Qualitative Traits in Foxtail Millet Resources.

Trait	Frequency Distribution						
	1	2	3	4	5	6	7
Seedling leaf color/SLC	0.7783	0.0936	0.1281	—	—	—	—
Leaf sheath color/LSC	0.9113	0.0345	0.0542	—	—	—	—
Seedling leaf posture/SLP	0.0443	0.5714	0.3498	0.0345	—	—	—
Leaf posture during flowering/LPDF	0.0640	0.0690	0.3596	0.5074	—	—	—
Neck shape/NS	0.0739	0.0887	0.7488	0.0887	—	—	—
Panicle pattern/TP	—	0.8325	0.0985	0.0640	—	0.0049	—
Panicle elasticity/SE	0.0837	0.7635	0.1527	—	—	—	—
Grain color/GC	0.1133	0.8473	—	0.0148	—	0.0197	0.0049

**Table 3:** Phenotypic Variation and Diversity of 13 Quantitative Traits in the Experimental Materials. Values are mean ± SD of the two-year accession means (n = 203). H′ was calculated with 10 classes (Section 2.4.1). Yield is expressed in kg·ha^−1^ (converted from kg·mu^−1^; 1 mu = 1/15 ha).

Trait	Mean ± SD	Range	CV (%)	Range Width (Max − Min)	Phenotypic Diversity Index (H′)
Growth Period (GP/d)	107.10 ± 9.18	84.24~134.51	8.57	50.27	2.049
Plant Height (PH/cm)	183.04 ± 13.39	146.11~224.73	7.32	78.61	2.039
Main Stem Diameter (MSD/mm)	7.82 ± 0.92	4.68~9.87	11.72	5.19	2.079
Number of Main Stem Nodes (NMSN)	13.03 ± 1.46	7.73~16.56	11.20	8.83	2.022
Leaf Length (LL/cm)	40.65 ± 6.97	29.33~113.16	17.16	83.83	1.697
Leaf Width (LW/cm)	2.29 ± 0.31	1.40~3.04	13.73	1.64	2.075
Panicle Length (EL/cm)	21.92 ± 5.42	11.25~78.24	24.72	66.99	1.736
Panicle Diameter(ED/mm)	23.26 ± 4.73	12.29~51.44	20.35	39.14	1.974
Thousand Grain Weight (TGW/g)	2.81 ± 0.26	2.22~3.58	9.24	1.35	2.066
Single Panicle Weight (SEW/g)	16.85 ± 6.34	3.54~46.85	37.65	43.31	2.000
Grain Weight per Panicle (GWE/g)	12.17 ± 4.74	2.19~34.31	38.91	32.13	2.027
Yield (Y/kg·ha^−1^)	3545.85 ± 1323.08	589.78~7387.32	37.31	6797.54	2.066
Dry Matter Accumulation (DMA/g)	26.65 ± 7.23	9.56~48.74	27.13	39.17	2.090

**Table 4:** Principal component analysis based on quantitative traits.

Trait	PC1	PC2	PC3	PC4	PC5
Growth Period	0.016	−0.021	0.154	0.576	0.364
Plant Height	0.360	−0.468	0.064	−0.387	0.166
Panicle Length	0.167	−0.121	0.757	0.037	−0.309
Panicle Diameter	0.562	−0.098	−0.087	0.208	−0.220
Main Stem Diameter	0.677	−0.417	0.011	0.009	−0.127
Number of Main Stem Nodes	0.632	−0.503	−0.105	−0.148	0.043
Leaf Length	0.206	−0.262	0.413	0.474	0.075
Leaf Width	0.293	0.160	−0.311	0.309	0.425
Grain Weight per Panicle	0.657	0.694	0.117	−0.039	−0.103
Thousand Grain Weight	−0.041	0.156	0.490	−0.382	0.647
Dry Matter Accumulation	0.770	−0.048	−0.184	−0.049	0.235
Yield	0.593	0.741	0.079	−0.075	−0.097
Eigenvalue	2.837	1.823	1.180	1.024	1.002
Contribution rate	23.645	15.188	9.832	8.532	8.351
Cumulative contribution rate	23.645	38.833	48.665	57.198	65.548

**Table 5:** Mean ± SD of the quantitative traits in the four groups defined by UPGMA cluster analysis (yield in kg·ha^−1^; clustering used yield in kg·mu^−1^; 1 kg·mu^−1^ = 15 kg·ha^−1^). F is the one-way ANOVA statistic across groups and is descriptive because the groups were formed from the same traits (*, **, *** *p* < 0.05, 0.01, 0.001; ns, not significant).

Trait	Group I (n = 129)	Group II (n = 37)	Group III (n = 32)	Group IV (n = 5)	F (*p*)
Number of accessions	129	37	32	5	
Growth period (GP, d)	106.34 ± 9.21	107.22 ± 7.06	107.81 ± 10.90	104.13 ± 3.04	0.4 ns
Plant height (PH, cm)	183.78 ± 12.73	182.72 ± 16.72	181.22 ± 12.54	178.12 ± 7.71	0.6 ns
Panicle length (EL, cm)	21.91 ± 6.30	21.13 ± 3.56	22.89 ± 3.14	21.75 ± 2.91	0.6 ns
Panicle diameter (ED, mm)	23.26 ± 4.25	21.60 ± 4.99	24.56 ± 3.51	23.52 ± 4.43	2.8 *
Main stem diameter (MSD, mm)	7.43 ± 0.86	7.05 ± 0.99	7.07 ± 0.97	8.39 ± 0.93	4.9 **
No. main stem nodes (NMSN)	13.20 ± 1.39	12.59 ± 1.97	12.78 ± 1.19	12.83 ± 0.99	2.0 ns
Leaf length (LL, cm)	40.58 ± 5.09	41.09 ± 13.04	40.83 ± 3.17	37.84 ± 2.24	0.3 ns
Leaf width (LW, cm)	2.32 ± 0.30	2.21 ± 0.31	2.34 ± 0.36	2.35 ± 0.16	1.4 ns
Single panicle weight (SEW, g)	16.88 ± 3.50	8.37 ± 2.07	23.90 ± 2.71	29.57 ± 1.99	170.5 ***
Grain weight per panicle (GWE, g)	11.30 ± 2.48	4.85 ± 1.49	16.21 ± 1.97	21.13 ± 1.98	184.9 ***
Thousand grain weight (TGW, g)	2.56 ± 0.21	2.57 ± 0.20	2.62 ± 0.21	2.63 ± 0.23	0.9 ns
Dry matter accumulation (DMA, g)	27.12 ± 6.88	21.66 ± 7.06	29.88 ± 6.06	33.35 ± 4.84	11.0 ***
Yield (Y, kg·ha^−1^)	3532 ± 670	1650 ± 414	5282 ± 460	6814 ± 381	265.1 ***

**Table 6:** Analysis of variance (ANOVA) and entry-mean repeatability (R) for 13 quantitative traits of 203 foxtail millet germplasm accessions evaluated in 2023 and 2024 (one accession mean per year; total df = 405; yield in kg·ha^−1^, converted from kg·mu^−1^, 1 mu = 1/15 ha). G × Y (residual) is the interaction mean square, confounded with error and used as the error term; R = 1 − MS(G × Y)/MS(G). * and *** indicate *p <* 0.05 and 0.001.

Trait	Source of Variation	df	SS	MS	F	Sig.	R (%)
**Growth Period (d)**	Genotype (G)	202	34,048.206	168.555	19.49	***	94.9%
	Year (E)	1	9835.682	9835.682	1137.39	***	
	*G × Y (residual)*	202	1746.818	8.648	—		
	Total	405	45,630.706	—	—		
**Plant Height (cm)**	Genotype (G)	202	72,444.207	358.635	94.01	***	98.9%
	Year (E)	1	2935.167	2935.167	769.43	***	
	*G × Y (residual)*	202	770.572	3.815	—		
	Total	405	76,149.946	—	—		
**Panicle Length (cm)**	Genotype (G)	202	11,858.215	58.704	334.43	***	99.7%
	Year (E)	1	108.712	108.712	619.31	***	
	*G × Y (residual)*	202	35.458	0.176	—		
	Total	405	12,002.385	—	—		
**Panicle Diameter (mm)**	Genotype (G)	202	9051.661	44.810	303.65	***	99.7%
	Year (E)	1	91.167	91.167	617.77	***	
	*G × Y (residual)*	202	29.810	0.148	—		
	Total	405	9172.638	—	—		
**Main Stem Diameter (mm)**	Genotype (G)	202	339.209	1.679	41.91	***	97.6%
	Year (E)	1	26.889	26.889	671.02	***	
	*G × Y (residual)*	202	8.094	0.04	—		
	Total	405	374.192	—	—		
**No. Main Stem Nodes**	Genotype (G)	202	859.100	4.253	96.85	***	99.0%
	Year (E)	1	30.942	30.942	704.65	***	
	*G × Y (residual)*	202	8.870	0.044	—		
	Total	405	898.912	—	—		
**Leaf Length (cm)**	Genotype (G)	202	19,645.403	97.254	264.16	***	99.6%
	Year (E)	1	234.372	234.372	636.59	***	
	*G × Y (residual)*	202	74.370	0.368	—		
	Total	405	19,954.144	—	—		
**Leaf Width (cm)**	Genotype (G)	202	39.77	0.197	4.78	***	79.1%
	Year (E)	1	25.129	25.129	610.57	***	
	*G × Y (residual)*	202	8.314	0.041	—		
	Total	405	73.213	—	—		
**Single Panicle** **Weight (g)**	Genotype (G)	202	16,256.062	80.476	283.09	***	99.6%
	Year (E)	1	37.086	37.086	130.46	***	
	*G × Y (residual)*	202	57.423	0.284	—		
	Total	405	16,350.571	—	—		
**Grain Weight** **per Panicle (g)**	Genotype (G)	202	9063.983	44.871	993.28	***	99.9%
	Year (E)	1	22.012	22.012	487.27	***	
	*G × Y (residual)*	202	9.125	0.045	—		
	Total	405	9095.121	—	—		
**Thousand Grain** **Weight (g)**	Genotype (G)	202	27.214	0.135	3.58	***	72.1%
	Year (E)	1	32.675	32.675	869.45	***	
	*G × Y (residual)*	202	7.592	0.038	—		
	Total	405	67.481	—	—		
**Dry Matter Accum. per Plant (g)**	Genotype (G)	202	21,113.934	104.524	1968.89	***	99.9%
	Year (E)	1	35.154	35.154	662.18	***	
	*G × Y (residual)* *	202	10.724	0.053	—		
	Total	405	21,159.812	—	—		
**Yield (kg·ha^−1^)**	Genotype (G)	202	1,435,364,589.1	7,105,765.3	7279.76	***	99.99%
	Year (E)	1	510,642.0	510,642.0	523.15	***	
	*G × Y (residual)* *	202	197,172.0	976.1	—		
	Total	405	1,436,072,402.9	—	—		

## Data Availability

The data presented in this study are available in the Supplementary Materials.

## Supplementary Materials

The following supporting information can be downloaded at: https://www.mdpi.com/article/10.3390/plants15203158/s1, Supplementary Table S1: Catalog of 203 foxtail millet germplasm accessions evaluated in northern Xinjiang (2023–2024), with germplasm status, province/region of origin, and acquisition source where available. Supplementary Table S2: Genotype × Environment (Year) interaction: mean values of 13 quantitative traits for 203 foxtail millet germplasm accessions, 2023 vs. 2024. Supplementary Table S3A: Previous crop and field management dates, 2023 and 2024 growing seasons. Supplementary Table S3B: Monthly meteorological conditions and irrigation volume at the experimental site, 2023 and 2024 growing seasons.
